# Beyond the Beat, Next-Generation Sequencing Discovery of Novel *RYR2* Gene Variant in Long QT Syndrome

**DOI:** 10.1155/crp/1928541

**Published:** 2025-08-27

**Authors:** Samira Kalayinia, Amir Ghaffari Jolfayi, Amirali Soheili, Majid Maleki, Mohammad Dalili, Mahdieh Soveizi, Saba Komijani

**Affiliations:** ^1^Cardiogenetic Research Center, Rajaie Cardiovascular Institute, Tehran, Iran; ^2^Cardiovascular Research Center, Rajaie Cardiovascular Institute, Tehran, Iran

**Keywords:** long QT syndrome, *RYR2*, variant, whole-exome sequencing

## Abstract

**Background:** Long QT syndrome (LQTS) is an inherited cardiac channelopathy marked by QT interval prolongation and increased risk of life-threatening arrhythmias. While variants in *KCNQ1*, *KCNH2*, and *SCN5A* explain most cases, many remain genetically unexplained. This study emphasizes the value of genetic testing in diagnosis and individualized therapy.

**Methods:** A 9-year-old boy with recurrent syncope was evaluated for LQTS. Clinical workup included history, physical exam, ECG, echocardiography, exercise testing, electrophysiology studies (EPS), Holter monitoring, and cardiac MRI. Family history was assessed. Genetic testing involved whole-exome sequencing (WES) and Sanger confirmation, followed by bioinformatic pathogenicity analysis.

**Results:** The boy's ECG showed a QTc of 470 ms, extending to 500 ms during EPS. No structural cardiac defects were detected. WES revealed a heterozygous missense variant, NM_001035.2:c.12370A > C (p.Ser4124Arg), in the *RYR2* gene. *In silico* tools predicted it to be pathogenic, and Sanger sequencing confirmed it. The variant was also identified in the patient's mother, who had a history of syncope, but not in the father. The patient responded well to propranolol and remained symptom-free for 18 months.

**Conclusion:** Identification of a pathogenic *RYR2* variant expands the known genetic spectrum of LQTS. The patient's clinical and familial findings highlight the need to consider *RYR2* in genetic testing panels, especially for atypical LQTS cases. Continued research is essential to further clarify the genetics of LQTS and guide targeted management.

## 1. Introduction

Long QT syndrome (LQTS) represents a group of inherited cardiac channelopathies characterized by the prolongation of the QT interval on electrocardiogram (ECG), predisposing affected individuals to life-threatening arrhythmias such as torsade's de pointes and sudden cardiac death (SCD) [1, 2]. Despite significant strides in understanding the genetic basis of LQTS and role of some genes like *KCNQ1, KCNH2*, and *SCN5A* in the 75% of the disorder, there still remains a considerable portion of cases where the underlying molecular mechanisms remain elusive [3]. Previous studies demonstrated that ryanodine receptor 2 (*RYR2*) gene is located in 1q42-q43 location of chromosome 1 and encodes an amino acid sequence for ryanodine receptor which can found in cardiac muscle sarcoplasmic reticulum (SR) [4]. It was demonstrated that mutation in this gene is associated with catecholaminergic polymorphic ventricular tachycardia (CPVT) [5], and even SCD [6]. *RYR2* is a critical component of cardiac excitation-contraction coupling and regulates calcium release [7, 8]. While primarily associated with CPVT [9], recent evidence suggests a potential role of *RYR2* in certain cases of LQTS, particularly those with overlapping phenotypes or atypical presentations [10]. In this research, we highlight the intricate interplay between *RYR2* and LQTS and present the symptoms suggestive of LQTS, including episodes of syncope and documented QT prolongation on ECG. We aim to broaden our understanding of the molecular underpinnings of this complex cardiac disorder. Furthermore, this case underscores the importance of integrating genetic testing into the clinical management of LQTS, facilitating accurate diagnosis, risk stratification, and personalized therapeutic interventions.

## 2. Methods and Materials

### 2.1. Clinical Presentation

A 9-year-old boy was admitted to the hospital due to frequent episodes of syncope. The patient was recruited for the study based on his clinical presentation suggestive of cardiac arrhythmia.

### 2.2. Clinical and Paraclinical Assessments

The patient underwent a comprehensive clinical evaluation, including a detailed medical history and physical examination. Additionally, paraclinical investigations such as ECG, echocardiography, and exercise testing were performed to assess cardiac function and identify any abnormalities.

### 2.3. Family History Assessment

A thorough assessment of the patient's family history was conducted to identify any instances of SCD or cardiovascular disorders among first-degree relatives.

### 2.4. Diagnostic Procedures

Electrophysiology studies (EPSs) were conducted to evaluate the patient's cardiac electrophysiological parameters. Holter monitoring and additional exercise testing were also performed to monitor cardiac rhythm and response to physical exertion. Finally, genetic evaluation to identify the genetic alternation which can lead to long QT is performed.

### 2.5. Magnetic Resonance Imaging (MRI)

Patient underwent cardiac magnetic resonance (CMR) imaging to identify the structural abnormalities.

### 2.6. Sample Collection and Whole-Exome Sequencing

Consent was obtained from the proband and all available family members, who then provided blood samples. Genomic DNA was extracted from the blood using standard salting-out methods following the manufacturer's instructions. WES was conducted on the proband. Exome capture was performed using an Agilent Sure Select All Exon V7 kit, and library sequencing was carried out using the Illumina HiSeq 6000 platform. An in-house bioinformatics pipeline was utilized, encompassing quality assessments, alignment to the human reference genome (GRCh37/hg19), variant calling, and annotation. Variants with minor allele frequencies (MAFs) below 1% in the 1000 Genomes Project, gnomAD, and Iranome were included in the analysis. Variants classified as likely pathogenic or pathogenic in ClinVar and the Human Gene Mutation Database (HGMD), and showing a relevant phenotype, were given precedence. The remaining variants underwent bioinformatics analysis using online tools such as MutationTaster, Polymorphism Phenotyping v2 (PolyPhen-2), Protein Variation Effect Analyzer (PROVEAN), Sorting Intolerant From Tolerant (SIFT), REVEL (Rare Exome Variant Ensemble Learner), and Combined Annotation-Dependent Depletion (CADD) to predict their impact on protein structure and function.

### 2.7. Polymerase Chain Reaction and Sanger Sequencing

Primer sequences flanking the potential variant were designed using the GenRunner. PCR amplification was conducted utilizing the SimpliAmp Thermal Cycler (Thermo Fisher Scientific) with 10 pmol/L of primers (forward primer: GGGACATATCCTTGATTCAGATG and reverse primer: AAAGATGTCCGAAAGAAGGTCA), 100 ng of DNA, 200 mmol/L of dNTP, 1.5 mmol/L of MgCl2, and 1 U of Taq DNA polymerase (Amplicon). The PCR protocol consisted of initial denaturation at 95°C for 5 min, followed by 35 cycles (30 s at 95°C, 30 s at 59°C, and 30 s at 72°C). Subsequently, the PCR products were subjected to sequencing using the ABI Sequencer 3500XL PE (Applied Biosystems, USA), and the data were analyzed with Codon Code Aligner software (CodonCode Corp, USA). Data collected from clinical evaluations, diagnostic procedures, and treatment interventions were analyzed descriptively to characterize the patient's clinical course and treatment response.

### 2.8. Ethical Considerations

This study was conducted in accordance with the principles outlined in the Declaration of Helsinki. Ethical approval was obtained from the Ethics Committees of Rajaie Cardiovascular Institute, Tehran, Iran (IR.RHC.REC.1402.003). Informed consent was obtained from the patient's legal guardians prior to participation in the study. Confidentiality of patient information was strictly maintained throughout the study period.

## 3. Results

### 3.1. Clinical Manifestations

**Figure 1:**
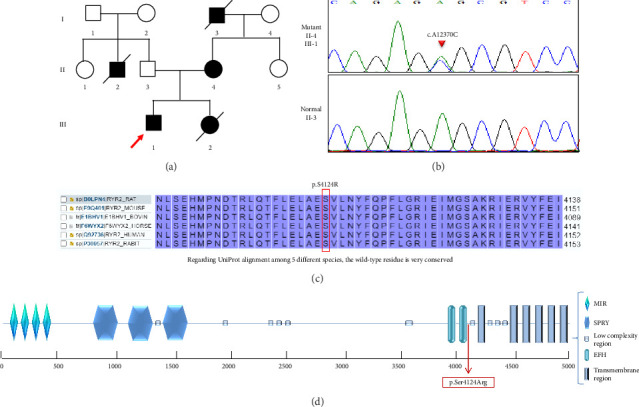
Family pedigree and genetic characterization of the *RYR2* p.Ser4124Arg variant. (a) Pedigree of the family showing the segregation of the heterozygous *RYR2* variant c.12370A > C (p.Ser4124Arg) in the affected proband and his mother. The proband's deceased sister is indicated, though no genetic data were available. (b) Sanger sequencing chromatograms showing the heterozygous A > C substitution in the proband (top panel) compared with a wild-type sequence (bottom panel). The position of the variant is highlighted by a black arrow. (c) Multiple sequence alignment of RYR2 orthologs from five species (human, mouse, rat, dog, and zebrafish) demonstrates that the serine residue at position 4124 is highly conserved across evolution, supporting the functional importance of this site. (d) Schematic representation of the RYR2 protein structure based on UniProt, indicating major functional domains. The p.Ser4124Arg variant is located within the central domain (residues ∼3778–4959), which is known to harbor disease-associated mutations.

A 9-year-old boy was admitted due to frequent episodes of syncope. These episodes occurred without warning signs and typically followed periods of physical exertion. The patient had a family history of SCD in his 15-year-old sister, and his mother has a history of syncope and transient loss of consciousness under stressful conditions (Figure 1). Routine laboratory investigations were conducted in both the proband and his mother to exclude reversible causes of QT prolongation. Serum levels of potassium, calcium, and magnesium were all within normal reference ranges. Thyroid function tests, including TSH and free T4, were also normal in both individuals. These findings support the conclusion that the observed QTc prolongation was not attributable to electrolyte imbalance or thyroid dysfunction.

### 3.2. Cardiac Evaluation

**Figure 2:**
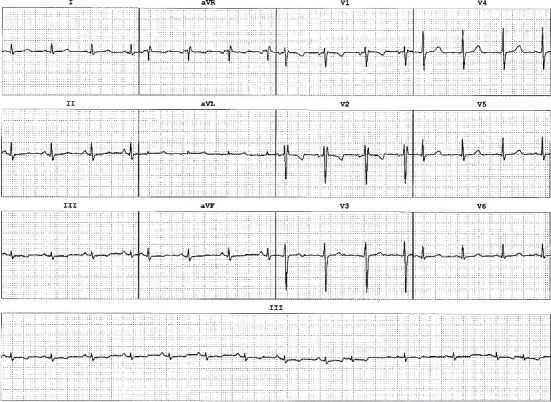
Representative electrocardiographic tracings obtained during the electrophysiology study (EPS) of the proband. Twelve-lead surface ECG recorded at baseline sinus rhythm demonstrates a prolonged corrected QT interval (QTc ≈ 500 ms, Bazett formula) with a normal PR interval and narrow QRS complexes. The QT interval was measured from the onset of the QRS to the end of the T-wave (tangent method) and averaged over three consecutive beats in lead II (arrows). No premature ventricular complexes or ventricular ectopy were observed during this recording. Heart rate at the time of acquisition was 72 bpm. These findings corroborate the diagnosis of long QT phenotype in the absence of structural cardiac abnormalities.

Figure 2 presents the ECG sample during the electrophysiological study of the 9-year-old boy who presented with frequent episodes of syncope. The ECG reveals a normal sinus rhythm, normal QRS axis, and QTc intervals of approximately 470 ms. Echocardiography revealed all parameters within normal ranges, with an ejection fraction (EF) of 60%. During a pediatric exercise test conducted to maximum effort, the patient exhibited normal tolerance and a normal chronotropic response. There were no significant ST-T changes, and the baseline ECG remained in normal sinus rhythm. Four minutes postexercise, the QTc interval was about 425 ms. According to the insufficient data for the diagnosis, and regarding the high-risk family history, the patient had undergone an EPS. During the EPS, the QTc was measured as prolonged as 500 ms. No further pathologic finding and no inducible arrhythmia was observed during the conventional EPS protocols. The patient was initiated on treatment with propranolol. The case has followed as outpatient for 18 months without any further symptom.

### 3.3. Imaging Findings

**Figure 3:**
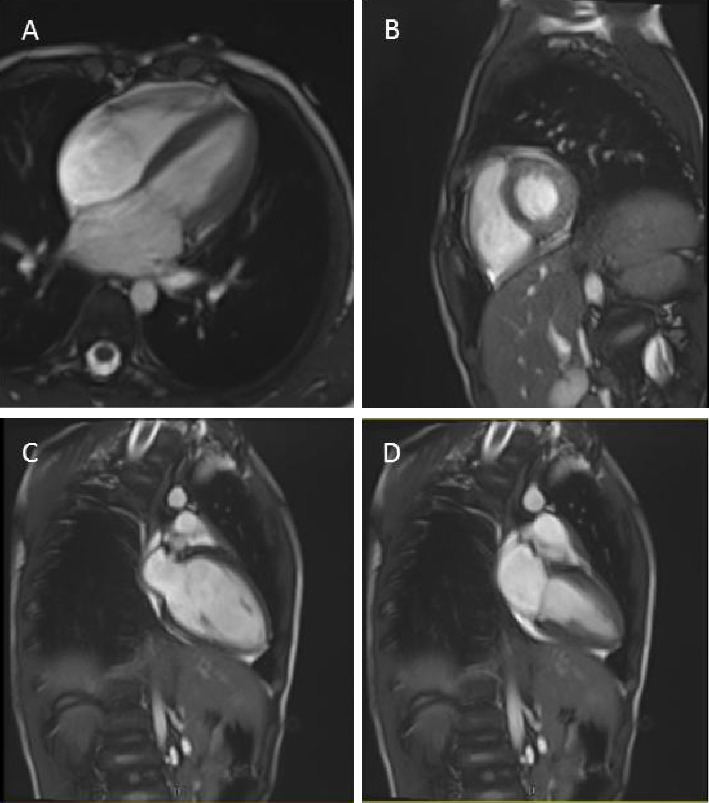
Cardiac magnetic resonance imaging (MRI) of the proband. Cardiac MRI demonstrated normal biventricular size and function with no evidence of structural abnormalities. (A) Four-chamber view at end-diastole showing normal chamber dimensions and myocardial wall thickness. (B) Short-axis view illustrating preserved left ventricular geometry. (C) End-diastolic vertical long-axis view showing intact ventricular walls and no signs of trabeculation or fibrosis. (D) End-systolic vertical long-axis view confirming normal systolic contraction. No late gadolinium enhancement or myocardial edema was observed. Overall, findings support the absence of cardiomyopathy or other structural heart disease in the proband.

Cardiovascular MRI was conducted using a myocarditis protocol, revealing normal cardiac morphology and function. Both the left and right atria were within normal size ranges, with respective areas of 12 cm^2^ and 11 cm^2^. Assessment of cardiac function indicated normal left ventricular (LV) and right ventricular (RV) sizes without hypertrophy, along with normal systolic function. The LV EF (LVEF) was measured at 64%. Similarly, the RVEF was 55%. Thoracic cage magnetic resonance angiography (MRA) with myocardial assessment showed normal dimensions of the thoracic aorta and pulmonary arteries. The thoracic aorta measured 14 mm, while the main pulmonary artery (MPA) measured 14 mm, with the right pulmonary artery (RPA) at 8 mm and the left pulmonary artery (LPA) at 10 mm. Furthermore, the gadolinium study reveals no evidence of myocardial inflammation or scar tissue on postcontrast imaging. The signal intensity ratio of myocardium over skeletal muscle is within the normal range. Overall, the findings suggest a normal cardiac morphology and function in a 9-year-old boy, and there were no significant structural abnormalities, hypertrophy, or dysfunction observed in either ventricle, and no evidence of myocardial inflammation or scar formation (Figure 3).

### 3.4. Genetic Findings

WES in the index identified a heterozygous substitution missense variant, NM_001035.2:c.12370A > C (p.Ser4124Arg) in the *RYR2* gene. This alternation results in change of the amino acid Serine to Arginine at position 4124. *In-silico* analysis by various software such as CADD (Phred score: 25.8), PolyPhen-2 (Probably damaging), SIFT and PROVEAN (Damaging), REVEL (score: 0.78), and MutationTaster (Disease causing) predicted that this change would be damaging. Moreover, according to the ACMG classification [11], this variant is classified pathogenic. Upon considering the ClinVar entry rs771994461 (Variation ID: 201330), which reports the same amino acid substitution caused by a different nucleotide change and is classified as pathogenic, the PS1 (Pathogenic Strong) criterion was applied. Consequently, the variant now meets the following criteria: PS1 (*strong*), PM1 (*moderate*), PM2 (*moderate*), PP1 (*moderate*), and PP3 (*supporting*), leading to a classification of pathogenic. Sanger sequencing in patient and in his family confirmed the variant, his father showed the wild type, and his mother showed variant in this position. Unfortunately, no DNA sample was available from the proband's deceased sister, who reportedly died suddenly in early childhood, precluding further segregation analysis. In addition, no extended family members were available or consented to testing. Therefore, while the observed inheritance pattern suggests possible cosegregation, the available data provide only limited support for this association and were accordingly considered as supporting-level evidence (PP1_Supporting) under ACMG/AMP guidelines. Regarding UniProt alignment among 5 different species, the wild-type residue was very conserved (Figure 1). No rare variants (MAF < 0.01) were identified in other genes known to be associated with LQTS, including *KCNQ1*, *KCNH2*, and *SCN5A*, and others included in established clinical gene panels, supporting the likelihood that the identified *RYR2* variant is the primary genetic contributor to the phenotype.

## 4. Discussion

In this study, we investigated a family of patient owning a 9-year-old boy with frequent syncope episodes, a family history of SCD in his sister, and normal initial clinical and imaging evaluations. Despite unremarkable ECGs, echocardiography, and cardiovascular MRI findings, an EPS of exercise test revealed a prolonged QTc interval. WES identified a pathogenic heterozygous substitution missense variant c.12370A > C (p.Ser4124Arg) in the *RYR2* gene, resulting in a change of the amino acid Serine to Arginine at position 4124, confirmed through Sanger sequencing. Cardiac MRI was performed exclusively on the proband, providing valuable structural and functional cardiac data. Unfortunately, the mother, who carries the same *RYR2* variant and has a history of syncope, did not undergo cardiac MRI due to personal and logistical reasons. The absence of imaging data from this genotype-positive family member limits our ability to detect subclinical myocardial abnormalities or structural contributors to the phenotype. Future evaluations including cardiac MRI of symptomatic family members are warranted to better understand the full spectrum of cardiac involvement associated with *RYR2* variants. The patient was treated with propranolol and has been symptom-free during follow-up.

A limitation of the present study is the lack of comprehensive copy number variant (CNV) analysis for major channelopathy-associated genes. Although WES was performed to identify single nucleotide variants and small indels, dedicated CNV detection methods were not applied. Given that deletions or duplications in genes linked to LQTS and CPVT can contribute to disease etiology, future studies should incorporate CNV analysis to provide a more complete genetic assessment. This approach would enhance the sensitivity of genetic testing and may uncover additional pathogenic variants missed by standard sequencing methods.

### 4.1. Mechanisms of *RYR2* Dysfunction in LQTS

A malfunctioning *RYR2* leads to a leakage of diastolic Ca^2+^ from the SR, contributing to delayed after-depolarizations which it is believed to be the underlying cause of fatal arrhythmias in heart failure and CPVT [6]. Various mechanisms have been proposed to explain dysfunctional *RYR2* in HF and CPVT, including increased phosphorylation, altered regulation, and disrupted interactions within the channel. *RYR2* alternation linked to LQTS influences the calcium handling in cardiac cells, leading to alterations in the cardiac action potential. These variants increase the sensitivity of the *RYR2* channel to luminal calcium, potentially causing abnormal electrical activities and arrhythmias in the heart. Experimental studies in mouse models and cell lines have shown that these variants can prolong the action potential duration, mimicking LQTS characteristics [12, 13]. This review examines *RYR2* dysfunction in terms of channel structure and function modulation and explores potential therapeutic approaches to stabilize *RYR2* function in heart disease [6].

### 4.2. Clinical Manifestations of LQTS With Etiology of *RYR2*

The clinical manifestations of LQTS associated with *RYR2* variants include several specific symptoms and conditions that reflect the underlying disturbances in cardiac electrophysiology. Key manifestations are mentioned in the following. Exercise or stress-induced arrhythmias can be observed in the presence of *RYR2* variants that often experience arrhythmias that are triggered by physical or emotional stress, not typically associated with the classic prolonged QT intervals seen on an ECG. Instead, these are due to abnormal calcium handling in the heart muscle, leading to ventricular tachycardia that can resemble LQTS during episodes of stress or exercise [10, 14]. Also, episodes of fainting or syncope are common and often triggered by physical activity or emotional stress, reflecting transient episodes of arrhythmia that can mimic other conditions like epilepsy [13]. SCD is an unfavorable outcome in the presence of *RYR2* variants. This variant can be considered as potential risk factor for SCD, particularly under conditions of exertion or stress, due to severe arrhythmias such as ventricular fibrillation or polymorphic ventricular tachycardia [15]. Unlike typical LQTS where a prolonged QT interval is a hallmark, *RYR2*-linked cases might not always show this feature prominently. Instead, the diagnosis can be obscured by borderline or normal QT intervals with episodes of arrhythmia only becoming evident under stress or during exercise testing [10, 14]. These clinical manifestations highlight the need for careful evaluation and management strategies tailored to the unique characteristics of *RYR2*-associated LQTS, distinguishing it from more common forms of the disorder. The diagnostic criteria for LQTS associated with *RYR2* variants, especially when considering the complexity of *RYR2*-related arrhythmias, involve a combination of genetic testing and clinical assessment [16, 17]. The presence of symptoms such as exercise or stress-induced syncope, a family history of SCD, and documented ventricular arrhythmias underlie the importance of a thorough clinical evaluation. Given that *RYR2* variants may not always result in a clearly prolonged QT interval, additional diagnostic markers such as stress testing and ambulatory monitoring might be necessary to provoke and record arrhythmogenic episodes indicative of LQTS or CPVT [10]. While typical LQTS shows prolonged QT intervals, *RYR2* variants may show normal or slightly altered QTc intervals [13, 14]. Overall, diagnosis of LQTS associated with *RYR2* requires careful consideration of both genetic and clinical data to differentiate it from other similar arrhythmogenic disorders. Several studies recommend a multidisciplinary approach, including the use of advanced genetic testing technologies and detailed phenotypic characterization to ensure accurate diagnosis and differentiation from other similar conditions [16–20]. Within the C-terminal/channel domain of RYR2 (exon 90), the variant p.Glu4182Gln (c.12544G > C) has been classified as pathogenic in ClinVar and reported *de novo* in a patient with CPVT. Other nearby residues—including Asn4178Tyr/Ser, Glu4187Gln, and Leu4188Pro—harbor pathogenic variants also linked to CPVT, further emphasizing the functional importance of this region, despite no LP/P variants being detected within ±2-3 nucleotides of c.12370.

### 4.3. Genetic Basis and Patterns of *RYR2*-Related LQTS

The genetic basis and inheritance patterns of LQTS associated with *RYR2* variants primarily involve variants in the *RYR2* gene. This gene encodes the *RYR2*, a key calcium release channel in cardiac muscle cells, which is essential for proper cardiac function. Variants in *RYR2* disrupt the normal flow of calcium ions within the heart, leading to abnormal heart rhythms and the prolongation of the QT interval on an ECG, a hallmark of LQTS [13]. These genetic alterations in *RYR2* can result in a spectrum of arrhythmias which also follows an autosomal dominant (AD) inheritance pattern. The specific nature and location of the mutation within the gene can significantly influence the clinical presentation and severity of the disorder, highlighting the complexity of the genetic landscape in *RYR2*-related cardiac conditions [10]. Accurate genetic diagnosis and comprehensive screening are crucial for an effective management of affected individuals. This emphasizes the nuanced interplay between genetic mutations and clinical manifestations in *RYR2*-related cardiac conditions, necessitating a deep understanding of genetic variations and their impact on cardiac physiology [21]. A notable limitation of our study is the absence of functional validation for the *RYR2* c.12370A > C (p.Ser4124Arg) variant. While in silico analysis and familial segregation support a pathogenic role, no electrophysiological or molecular studies were performed to directly assess the impact of this amino acid substitution on *RYR2* channel activity. As this is a single-patient case report, such experiments were beyond the scope of the current work. However, we emphasize that future functional studies will be essential to confirm the mechanistic consequences of this variant and strengthen its clinical interpretation.

### 4.4. Management and Treatment Strategies for LQTS Caused by *RYR2* Variants

Management and treatment strategies for LQTS caused by *RYR2* variants are centered around preventing arrhythmic events, typically through medication, lifestyle adjustments, and in some cases, surgical interventions. Beta-blockers are commonly used to manage symptoms by reducing heart rate and minimizing stress-induced arrhythmias [15]. For cases where beta-blockers are insufficient, calcium channel blockers or sodium channel blockers like flecainide may be used. These medications help by stabilization of cardiac electrical activity and reduction of the incidence of arrhythmias. Additionally, lifestyle modifications, including avoiding strenuous exercise and stress management, are recommended to decrease the likelihood of triggering arrhythmias [22]. Pharmacological interventions often need to be complemented with careful monitoring and dose-adjustment. This might involve regular follow-up appointments, ECG tests, and possibly exercise stress tests to assess the effectiveness of the treatment and adjust it as needed. The specific choice of medication can be influenced by the particular characteristics of the *RYR2* mutation and the patient's response to initial treatments [23]. Genetic counseling is also recommended for patients and their families, as it provides information about inheritance patterns, risks to other family members, and implications for future generations. This is particularly important for conditions like LQTS where familial patterns are common, and early diagnosis can prevent severe outcomes [18, 24, 25]. Furthermore, Chou et al. [24] reviewed case series from Hong Kong on rare genetic mutations associated with LQTS, emphasizing the diversity of genetic variants that can influence the clinical management of the disorder. This study supports the need for extensive genetic screening to better understand and manage LQTS effectively. Considering recent studies, Mehta et al. [26] also identified a targeted and testable antiarrhythmic therapy for LQTS type 2 using a patient-specific cellular model. This research represents a significant step towards personalized medicine, suggesting that drugs like lumacaftor could potentially correct genetic defects in LQTS patients. More studies are required to test these possible antiarrhythmic medications. Although *RYR2* is not among the canonical genes implicated in classical LQTS, there is growing recognition of its involvement in atypical or overlapping arrhythmia phenotypes. Traditionally associated with CPVT, *RYR2* variants have also been reported in patients with borderline or prolonged QT intervals, suggesting potential modulatory effects on repolarization. In our case, the identified *RYR2* missense variant NM_001035.2:c.12370A > C (p.Ser4124Arg) was associated with QTc prolongation and a family history of syncope, without features typical of CPVT such as bidirectional VT or exercise-induced arrhythmias. This supports the hypothesis that certain *RYR2* variants may contribute to an atypical LQTS phenotype or a phenotypic continuum between LQTS and CPVT. Further functional studies and genotype-phenotype correlations are needed to delineate the precise role of *RYR2* in cardiac repolarization disorders [15, 27].

## 5. Conclusion

This study highlights the importance of comprehensive genetic testing in patients with unexplained syncope and a family history of SCD, even when initial clinical, electrophysiological, and imaging evaluations are normal. The identification of a pathogenic *RYR2* gene variant in our patient underscores the role of genetic factors in arrhythmogenic disorders. Early diagnosis and treatment with medications such as propranolol can be crucial in managing these patients and preventing potentially fatal events. Our findings suggest that incorporating genetic screening into the diagnostic protocol for similar cases could significantly improve patient outcomes.

## Data Availability

The data supporting the findings of this study are available from the corresponding author upon reasonable request.

## References

[1] Vohra J. (2007). The Long QT Syndrome. *Heart Lung & Circulation*.

[2] Viskin S. (1999). Long QT Syndromes and Torsade De Pointes. *The Lancet*.

[3] Tester D. J., Ackerman M. J. (2014). Genetics of Long QT Syndrome. *Methodist DeBakey cardiovascular journal*.

[4] Swan H., Piippo K., Viitasalo M. (1999). Arrhythmic Disorder Mapped to Chromosome 1q42–q43 Causes Malignant Polymorphic Ventricular Tachycardia in Structurally Normal Hearts. *Journal of the American College of Cardiology*.

[5] Priori S. G., Napolitano C., Tiso N. (2001). Mutations in the Cardiac Ryanodine Receptor Gene (Hryr2) Underlie Catecholaminergic Polymorphic Ventricular Tachycardia. *ACC Current Journal Review*.

[6] Blayney L. M., Lai F. A. (2009). Ryanodine Receptor-Mediated Arrhythmias and Sudden Cardiac Death. *Pharmacology & Therapeutics*.

[7] Guo Y., Cao Y., Jardin B. D. (2023). Ryanodine Receptor 2 (RYR2) Dysfunction Activates the Unfolded Protein Response and Perturbs Cardiomyocyte Maturation. *Cardiovascular Research*.

[8] Taur Y., Frishman W. H. (2005). The Cardiac Ryanodine Receptor (Ryr2) and Its Role in Heart Disease. *Cardiology in Review*.

[9] Dulhunty A. F. (2022). Molecular Changes in the Cardiac RyR2 With Catecholaminergic Polymorphic Ventricular Tachycardia (CPVT). *Frontiers in Physiology*.

[10] Tester D. J., Kopplin L. J., Will M. L., Ackerman M. J. (2005). Spectrum and Prevalence of Cardiac Ryanodine Receptor (Ryr2) Mutations in a Cohort of Unrelated Patients Referred Explicitly for Long QT Syndrome Genetic Testing. *Heart Rhythm*.

[11] Richards S., Aziz N., Bale S. (2015). Standards and Guidelines for the Interpretation of Sequence Variants: A Joint Consensus Recommendation of the American College of Medical Genetics and Genomics and the Association for Molecular Pathology. *Genetics in Medicine*.

[12] Kamga M. V. K., Reppel M., Hescheler J., Nguemo F. (2021). Modeling Genetic Cardiac Channelopathies Using Induced Pluripotent Stem Cells–Status Quo From an Electrophysiological Perspective. *Biochemical Pharmacology*.

[13] Cai W., Valdivia C. R., Li S. (2022). Abstract 11999: Molecular and Cellular Characterization of RyR2 Mutations Linked to Long-QT Syndrome. *Circulation*.

[14] Hasegawa K., Gao J., Ohno S. (2022). Oral Adrenergic Agents Produced Ventricular Fibrillation and QT Prolongation in an Elderly Patient Carrying an RYR2 Variant. *International Heart Journal*.

[15] Medeiros-Domingo A., Bhuiyan Z. A., Tester D. J. (2009). The RYR2-encoded Ryanodine Receptor/Calcium Release Channel in Patients Diagnosed Previously With Either Catecholaminergic Polymorphic Ventricular Tachycardia or Genotype Negative, Exercise-Induced Long QT Syndrome: A Comprehensive Open Reading Frame Mutational Analysis. *Journal of the American College of Cardiology*.

[16] Draelos R. L., Ezekian J. E., Zhuang F. (2022). GENESIS: Gene-Specific Machine Learning Models for Variants of Uncertain Significance Found in Catecholaminergic Polymorphic Ventricular Tachycardia and Long QT Syndrome-Associated Genes. *Circulation: Arrhythmia and Electrophysiology*.

[17] Letsas K. P., Prappa E., Bazoukis G. (2020). A Novel Variant of RyR2 Gene in a Family Misdiagnosed as Congenital Long QT Syndrome: The Importance of Genetic Testing. *Journal of Electrocardiology*.

[18] Nagata Y., Watanabe R., Eichhorn C. (2022). Targeted Deep Sequencing Analyses of Long QT Syndrome in a Japanese Population. *PLoS One*.

[19] Adler A., Novelli V., Amin A. S. (2020). An International, Multicentered, Evidence-Based Reappraisal of Genes Reported to Cause Congenital Long QT Syndrome. *Circulation*.

[20] Schwartz P. J., Moss A. J., Vincent G. M., Crampton R. S. (1993). Diagnostic Criteria for the Long QT Syndrome. an Update. *Circulation*.

[21] Moss A. J., Robinson J. L. (1992). The Long-QT Syndrome: Genetic Considerations. *Trends in Cardiovascular Medicine*.

[22] Khan I. A., Gowda R. M. (2004). Novel Therapeutics for Treatment of long-QT Syndrome and Torsade De Pointes. *International Journal of Cardiology*.

[23] Khan I. A. (2002). Long QT Syndrome: Diagnosis and Management. *American Heart Journal*.

[24] Chou O. H., Hui J. M., Lee Y. H. (2022). Rare Genetic Mutations Associated with Long QT Syndrome in Hong Kong Chinese Patients. *Annals of Clinical Cardiology*.

[25] Giudicessi J. R., Ackerman M. J. (2016). Calcium Revisited: New Insights Into the Molecular Basis of Long-QT Syndrome. *Circulation: Arrhythmia and Electrophysiology*.

[26] Mehta A., Ramachandra C. J., Singh P. (2018). Identification of a Targeted and Testable Antiarrhythmic Therapy for Long-QT Syndrome Type 2 Using a Patient-Specific Cellular Model. *European Heart Journal*.

[27] Bhuiyan Z. A., van den Berg M. P., van Tintelen J. P. (2007). Expanding Spectrum of Human RYR2-Related Disease: New Electrocardiographic, Structural, and Genetic Features. *Circulation*.

